# “Walking into the unknown…” key challenges of pregnancy and early parenting with inflammatory arthritis: a systematic review and thematic synthesis of qualitative studies

**DOI:** 10.1186/s13075-021-02493-z

**Published:** 2021-04-21

**Authors:** Nevena Rebić, Ria Garg, Ursula Ellis, Vanessa Kitchin, Sarah Munro, Glen Hazlewood, Neda Amiri, Nick Bansback, Mary A. De Vera

**Affiliations:** 1 Faculty of Pharmaceutical Sciences, University of British Columbia, 2405 Wesbrook Mall, Vancouver, BC V6T 1Z3 Canada; 2 Arthritis Research Centre of Canada, 5591 Number 3 Rd, Richmond, BC V6X 2C7 Canada; 3 Collaboration for Outcomes Research and Evaluation, 2405 Wesbrook Mall, Vancouver, BC V6T 1Z3 Canada; 4 University of British Columbia Library, 2198 Health Sciences Mall, Vancouver, BC V6T 1Z3 Canada; 5 Department of Obstetrics and Gynaecology, University of British Columbia, Suite 930, 1125 Howe Street, Vancouver, BC V6Z 2K8 Canada; 6 Centre for Health Evaluation and Outcome Sciences, 588–1081 Burrard Street St. Paul’s Hospital, Vancouver, BC V6Z 1Y6 Canada; 7 Cumming School of Medicine, University of Calgary, 3330 Hospital Drive NW, Calgary, Alberta T2N 4N1 Canada; 8 Division of Rheumatology, Department of Medicine, Faculty of Medicine, University of British Columbia, 802–1200 Burrard Street, Vancouver, BC V6Z 2C7 Canada; 9 School of Populations and Public Health, University of British Columbia, 2206 East Mall, Vancouver, BC V6T 1Z3 Canada

**Keywords:** Inflammatory arthritis, Pregnancy, Systematic review, Qualitative methods

## Abstract

**Background:**

To conduct a systematic review and thematic synthesis of qualitative studies on the pregnancy and early parenting experiences of patients with inflammatory arthritis (IA).

**Methods:**

We searched online databases for English-language, qualitative studies capturing the experiences of females with IA or their healthcare providers with pregnancy and/or early parenthood. We extracted findings from included studies and used thematic synthesis to develop descriptive and higher-order analytical themes.

**Results:**

Of 20 included studies, our analysis identified 5 analytical themes among patients and 3 among providers. Patients’ reproductive desires, the impact of IA on their ability to experience pregnancy, and the availability of information to guide preparedness informed their pregnancy decisions. Patients’ IA management, pregnancy expectations, and access to support influenced their reproductive experiences. Patients’ experiences seeking information and care revealed substantial gaps in reproductive care provision to patients with IA. Reproductive uncertainty related to IA placed a heavy burden on patients’ emotional and psychological wellbeing. Reproductive care provision was influenced by providers’ perceived professional responsibility to address patients’ reproductive goals, fears of negative outcomes, and capacity to harness patient trust, incorporate reproductive care into rheumatology practice and facilitate multi-disciplinary care coordination.

**Conclusions:**

Our review illuminated several barriers to experiencing pregnancy among patients with IA, particularly related to pregnancy planning support, availability of information, and care coordination among the patient’s healthcare team. To improve care, these barriers may be mitigated through the provision of relevant, practical, and consistent information as well as patient-centred multi-disciplinary approaches for managing pregnancy among patients with IA.

## Background

Inflammatory arthritides (IA), including rheumatoid arthritis (RA) and systematic lupus erythematosus (SLE), are chronic inflammatory diseases that commonly affect female patients of reproductive age [1, 2]. As disease activity and associated medication use may cause pregnancy complications, patients with IA face unique challenges related to planning and managing pregnancy [2].

The reproductive health needs of individuals with IA are often inadequately managed. In a 2012 international survey of female patients with chronic inflammatory conditions, including IA, the majority of respondents reported pregnancy was not sufficiently addressed in clinical practice and only 40% received consistent reproductive guidance from healthcare providers [3]. Indeed, there is a body of research indicating patients have substantial information needs about pregnancy and early parenting, particularly related to medication use [4–6]. Poor dissemination of medication information for pregnancy can lead patients to discontinue beneficial medications due to fear of foetal harm, which may result in poorer outcomes for both mother and baby related to active disease [7]. Given the complexity of managing pregnancy with IA, we aimed to systematically review the qualitative literature reporting patient and provider perspectives of reproductive care provision for female patients with IA.

## Methods

### Search strategy

We developed and refined our search strategy parameters in consultation with two health sciences librarians (UE, VK). We searched Ovid MEDLINE, Ovid EMBASE, CINAHL, PsycINFO via EBSCOHost, and Social Sciences Citation Index from inception to February 2020. Additionally, we conducted a hand search of relevant bibliographies. We mapped subject headings and keywords of unindexed terms related to IA, pregnancy and parenthood, and qualitative methods (see Additional file 1) to identify citations meeting our inclusion criteria: primary research article; study sample of female patients with IA (i.e., RA, SLE, ankylosing spondylitis [AS], juvenile idiopathic arthritis [JIA], psoriatic arthritis [PsA]) or their healthcare providers, qualitative study design capturing participants’ pregnancy and/or early parenthood experiences, and published in English. The enhancing transparency in reporting the synthesis of qualitative research (ENTREQ) statement guided our reporting [8].

### Study selection

Two authors (NR, RG) independently reviewed titles and abstracts of identified records. Studies meeting the inclusion criteria received full text review. Thereafter, those that continued to satisfy inclusion criteria were forwarded for quality assessment and systematic review. Disagreement was resolved by consensus. We extracted information on the publication year, country, diseases studied, participant characteristics (i.e,. age, sex, patient/provider), study objective, data collection, and analysis methods, reported reproductive topics.

### Quality assessment

We used the Critical Appraisal Skills Programme (CASP) Qualitative Research Checklist [9, 10] to assess the quality of included studies. This checklist comprises 10 questions assessing research aims, design, methodology, recruitment, data collection, data analysis, ethical issues, participant-researcher relationship, findings, and research value [9]. Two authors (NR, RG) independently evaluated studies and resolved disagreements through discussion.

### Synthesis

Included studies were imported into NVivo 12.6.0. We analysed study results data, including primary quotations and authors’ interpretations included in the text, tables, and supplementary materials. One author (NR) preformed the thematic synthesis, a method combining grounded theory and meta-ethnography approaches [11, 12]. This involved conducting line-by-line coding to develop an initial coding framework which was subsequently applied across all articles [11]. Similarities and differences between codes were used to inform their hierarchical grouping into descriptive themes [11]. Relationships within and between descriptive themes were analysed to generate analytical themes of higher-order constructs that addressed our review objectives [11]. Two authors (NR, MDV) reviewed the coding framework and discussed developing themes.

## Results

**Fig. 1:**
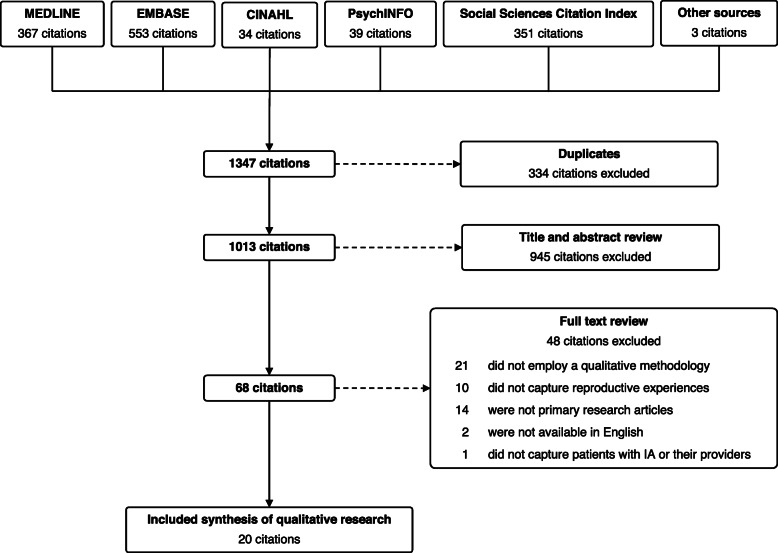
Selection of included studies

Our search (Fig. 1) identified 1347 articles. After exclusions, we included 20 studies for thematic analysis (Table 1). The majority (*n* = 19) reported on 368 female patient perspectives and 3 studies reported on 51 provider perspectives. With respect to disease, 14 studies limited inclusion to a single diagnosis (8 examined RA patients, 4 SLE, and 2 JIA) with remaining studies including a mix of IA patients. Studies reporting provider perspectives compromised primarily rheumatologists as well as midwives, general practitioners, nephrologists, and health visitors.

**Table 1:** Characteristics of studies included in systematic review

Author	Year	Country	Disease	Perspective	N	Age (mean, years)	Sex (%F)	Data collection and analysis	Aim	Reproductive topic		
										Pregnancy	Parenting	Medications
Lempp et al. [13]	2006	UK	RA	Patient	26	56	85	Individual semi-structured interviews, qualitative content analysis	To explore experiences of living with RA and its impact on patients’ private and public lives.	●	◐	●
Backman et al. [14]	2007	Canada	RA, AS, JIA, SLE	Patient	12	- (24, 53 range)	100	Individual semi-structured interviews, Grounded Theory	To describe the impact of IA on parenting and identify strategies used by mothers with IA.	○	●	○
Mitton et al. [15]	2007	UK	RA	Patient	7	36 (21, 41 range)	100	Individual semi-structured interviews, phenomenological analysis	To explore the health and life experiences of mothers with RA.	●	●	○
Östlie et al. [16]	2009	Norway	JIA	Patient	15	30 (22, 38 range)	60	Individual semi-structured interviews, qualitative content analysis	To understand living with JIA in childhood, adolescence, and adult life.	◐	◐	○
McElhone et al. [17]	2010	UK	SLE	Patient	30	49 (21, 75 range)	100	Individual semi-structured interviews, phenomenological analysis	To explore how SLE has impacted patients’ lives.	○	●	○
Eyckmans et al. [18]	2011	Belgium	JIA	Patient	11	24 (20, 30 range)	73	Individual semi-structured interviews, Grounded Theory	To examine growing up with JIA.	●	○	○
Kristiansen et al. [19]	2012	Denmark	RA	Patient	32	58 (31, 81 range)	59	Focus group interviews, qualitative content analysis	To explore how everyday life is affected by RA.	○	●	○
Meade et al. [20]	2012	Australia	RA	Patient	14	37 (25, 51 range)	100	Written accounts, thematic analysis	To examined female patients’ experiences negotiating their family decisions in the context of RA.	●	●	◐
Feldthusen et al. [21]	2013	Sweden	RA	Patient	25	46 (20, 60 range)	76	Focus group interviews, qualitative content analysis	To describe how individuals with RA of working age experience and handle their fatigue.	○	●	○
Ackerman et al. [4]	2015	Australia	RA	Patient	27	32 (31, 36 range)	100	Individual and focus group interviews, inductive analysis	To determine the need and preferred modes of delivering information regarding pregnancy, post-natal care, and early parenting among female patients with RA.	●	●	●
Nota et al. [22]	2015	Netherlands	IA (RA, AS, PsA)	Patient	32	54 (25, 82 range)	81	Individual semi-structured interviews, thematic analysis	To explore patients’ considerations when deciding whether to use DMARDs and their information needs for participating in the decision-making process.	◐	○	●
Ammerlaan et al. [23]	2017	Netherlands	RA, SpA, OA, FM, PsA, other^a^	Patients	35	42 (26, 74 range)	94	Online asynchronous focus group interview, content analysis	To investigate preferences and needs for the structure and content of a person-centred online self-management support intervention for patients with ARDs.	◐	●	○
Feddersen et al. [24]	2018	Denmark	RA	Patient	20		100	Individual interview and participant observation, Grounded Theory	To explore how female patients with RA manage their disease, motherhood, and work life.	○	●	○
Phillips et al. [5]	2018	UK	SLE, RA, JIA, PsA, other	Patient Provider^b^	22 7	34 (29, 39 range) NR	100	Individual narrative interviews, thematic analysis	To identify the information and support needs of women with ARDs during pregnancy planning, pregnancy, and early parenting.	●	●	●
Birru et al. [25]	2019	USA	n/a	Provider	12	NR	58	Individual semi-structured interview, thematic analysis	To assess rheumatologists’ perspectives, attitudes, and practices regarding pregnancy counseling and reproductive health care.	●	○	●
Clowse et al. [26]	2019	USA	SLE	Patient Provider	15 32	(21, 25 range) 44 (28, 67 range)	100 63	Focus group interviews, qualitative analysis	To identify factors influencing the management of SLE during pregnancy among community and university rheumatologists.	●	○	●
Gomez et al. [27]	2019	USA	SLE, other^c^	Patient	12^d^	21.5 (1.7 SD)	83	Individual semi-structured interviews, thematic analysis	To explore perspectives of family planning among young people who believe they are infertile or will have challenges carrying a pregnancy to term due to medical conditions or procedures.	●	○	○
Phuti et al. [28]	2019	South Africa	SLE	Patient	25	31 (22, 45 range)	100	Individual interview, thematic analysis	To explore living experiences, perceptions, and unmet needs of South African patients with SLE.	●	◐	○
Chew et al. [29]	2019	NR	RA	Patient	59^e^	NR	100	Written accounts, thematic analysis	To explore the information needs and concerns of female patients with RA related to pregnancy and parenting.	●	●	●
Phuti^f^ et al. [30]	2020	South Africa	SLE	Patient	25	31 (22, 45 range)	100	Individual interview, thematic analysis	To explore the perceptions and experiences of female patients with SLE related to fertility and pregnancy.	●	●	◐

“○”, not examined; “◐”, partially examined; “●” examined; *n/a*, not applicable; *NR*, not reported

^a^Includes systemic sclerosis and palindromic rheumatism

^b^Consistent of 2 consultant rheumatologists, 1 general practitioner, 1 nephrologist, 2 midwives, and 1 health visitor

^c^Includes ovarian cysts, polycystic ovary syndrome, endometriosis, low sperm count, intensive radiology treatment, complications from previous intrauterine device, and thyroid cancer

^d^Includes one female participant with lupus

^e^*N* is reported as the total number of online written accounted analysed in the article

^f^Uses same sample as Phuti et al. [28]

The CASP Checklist appraisal is summarized in Table 2. All studies clearly stated their aims, appropriately used a qualitative research methodology, and adequately justified its use. Most studies collected, analysed, and reported data adequately, used rigorous data analysis, and clearly stated research findings.

**Table 2:** Quality assessment of included studies

	Lempp et al. [13]	Backman et al. [14]	Mitton et al. [15]	Östlie et al. [16]	McElhone et al. [17]	Eyckmans et al. [18]	Kristiansen et al. [19]	Meade et al. [20]	Feldthusen et al. [21]	Ackerman et al. [4]	Nota et al. [22]	Ammerlaan et al. [23]	Feddersen et al. [24]	Phillips et al. [5]	Birru et al. [25]	Clowse et al. [26]	Gomez et al. [27]	Phuti et al. [28]	Chew et al. [29]	Phuti et al. [30]
**CASP Qualitative Research Checklist**																				
Clear statement of the aims of the research?	Yes	Yes	Yes	Yes	Yes	Yes	Yes	Yes	Yes	Yes	Yes	Yes	Yes	Yes	Yes	Yes	Yes	Yes	Yes	Yes
Was a qualitative methodology appropriate?	Yes	Yes	Yes	Yes	Yes	Yes	Yes	Yes	Yes	Yes	Yes	Yes	Yes	Yes	Yes	Yes	Yes	Yes	Yes	Yes
Was the research design appropriate for the aims?	Yes	Yes	Yes	Yes	Yes	Yes	Yes	Yes	Yes	Yes	Yes	Yes	Yes	Yes	Yes	Yes	Yes	Yes	Yes	Yes
Was the recruitment strategy appropriate for the aims?	Yes	Yes	Partly	Yes	Yes	Yes	Partly	Yes	Yes	Yes	Yes	Yes	Yes	Yes	Partial	Yes	Yes	Yes	Yes	Yes
Were the data collected in a way that addressed the research issue?	Yes	Yes	Yes	Yes	Yes	Yes	Yes	Yes	Yes	Yes	Yes	Partly	Yes	Yes	Yes	Yes	Partly	Yes	Yes	Yes
Has the relationship between researcher and participants been adequately considered?	No	No	No	Yes	No	Yes	Partly	No	No	No	No	No	No	No	No	No	No	Yes	No	Yes
Have ethical issues been taken into consideration?	Yes	Yes	Yes	Yes	Yes	Yes	Yes	Yes	Yes	Yes	Yes	Yes	Yes	Yes	Yes	Yes	Yes	Yes	Yes	Yes
Was data analysis sufficiently rigorous?	Partly	Yes	Yes	Yes	Yes	Yes	Partly	Yes	Yes	Yes	Yes	Partly	Yes	Yes	Yes	Yes	Yes	Yes	Yes	Yes
Is there a clear statement of findings?	Yes	Yes	Yes	Yes	Yes	Yes	Partly	Partly	Yes	Yes	Yes	Partly	Yes	Yes	Yes	Yes	Yes	Yes	Yes	Yes
How valuable is the research?	High	High	Medium	High	High	High	Medium	High	High	High	High	Medium	High	High	High	High	High	High	High	High

**Fig. 2:**
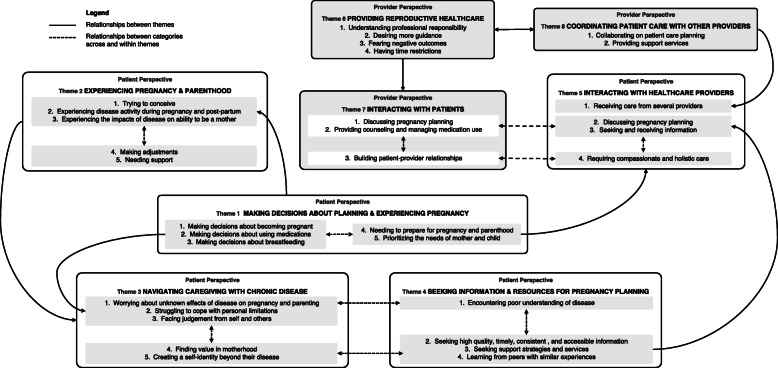
Thematic map of identified analytical and descriptive themes of patient and healthcare provider perspectives. Legend: Making decisions about planning and experiencing pregnancy (Theme 1) is related to experiences of pregnancy and parenting, which are shaped by patients’ disease experience, ability to make adjustments, and secure support (Theme 2). Together these impact pregnancy and parenting with chronic disease (Theme 3). Psychological hardships (Theme 3) are counteracted by expanding individual identity and disease knowledge (Theme 4). Collectively, Themes 1 and 4 influence patient-provider interactions (Theme 5). Providers’ knowledge, fears, and priorities (Theme 6) shape patients’ interactions (Theme 7) and care experiences (Theme 5). Multi-disciplinary care coordination (Theme 8) facilitated reproductive care delivery (Theme 5 and 6)

Our synthesis identified 5 analytical themes among patients: making decisions about planning and experiencing pregnancy, experiencing pregnancy and parenting, navigating caregiving with chronic disease, seeking information and resources for pregnancy planning, and interacting with healthcare providers. We additionally identified 3 analytical themes among providers: providing reproductive health care, interacting with patients, and coordinating patient care with other providers*.* Our coding framework and representative quotations are presented in Table 3. Relationships between analytical and descriptive themes are depicted in a conceptional model (Fig. 2).

**Table 3:** Topics covered and illustrative text excerpts for descriptive themes

Analytic theme	Descriptive theme	Topics covered	Example quotations	Ref.
**Patient perspectives**				
Making decisions about planning and experiencing pregnancy	Making decisions about becoming pregnant	• Factors impacting pregnancy decisions • Fear of IA impeding ability to raise a child • Childbearing alternatives (e.g., adoption) • Pregnancy prevention and termination	[Women] were careful in considering their capacity to manage pregnancies and motherhood. These considerations were important in their decision-making, and based on, as one participant commented: ‘...an honest appreciation of the reality of the situation’ which is unlike that of ‘other couples [who] get pregnant and then consider the consequences’.	[20]
			One woman, who expressed fear of not being able to take care of a child and therefore had chosen not to have children, confronted another participant in the group. ‘You had the courage to have children … despite this (fatigue). I know that it is not an option for me … where would I get the energy for that from?’	[21]
	Making decisions about using medications	• Factors impacting medication decisions • Wanting more information and decisional support about taking medications	Medication safety was an important topic, and members expressed concerns over the use of biologics and prednisone during pregnancy, indicating that they would rather not take medications during the pregnancy if there were even a slight risk to the foetus.	[29]
			‘But nobody’s told me what the side effects of steroids are during pregnancy, they just say it’s kind of the safest option really and that they’ll judge it when they get to it depending on how bad I am. So I’m walking into the unknown, I have no idea.’	[5]
	Making decisions about breastfeeding	• Factors impacting breastfeeding decisions • Wanting more information and decisional support about breastfeeding	‘By about 4 weeks my mum was saying please stop breastfeeding and she’d been very pro-breastfeeding, but she could obviously see I was struggling (with mobility and pain), but I marched on and then at 6 weeks I dropped the child.’	[20]
	Needing to prepare for pregnancy and parenthood	• Factors influencing conception and pregnancy planning • Parenting while managing post-partum flares • Establishing support systems (e.g., family members, mothers with IA)	The process of acceptance necessitated planning, as spontaneity regarding conception is not an option for women with RA. For some, it meant facing realities such as financial burdens: ‘...it is really important ... to have significant savings in the bank prior to falling pregnant. I did, which helped immensely because I was unable to continue the biologic medication, I was unable to work and had to rely on my savings to pay for medicine, doctors, etc.’	[20]
			[One participant], who had been pregnant once, described openness in her pregnancy timeline because of her Lupus diagnosis: ‘Yeah, so if I’m like 26, and they are like, yeah, you are in remission, and I’m in a good place with everything else, I figure that might be the time. So I would not want to wait necessarily in case I get sick again.’ Although [she] would prefer to wait until age 27 or 28 to become pregnant, she also recognized the need for flexibility in those plans because she could not predict the status of her lupus […] Because of this, she said, ‘Family planning for me would probably go around when I’m in remission.’	[27]
	Prioritizing the needs of mother and child	• Trade-offs between pregnancy/breastfeeding desires and potential harms of IA management • Self-advocacy	Another member described why, even after discussing the risks of pregnancy and the appropriate medications to take, women may move forward with their own pregnancy plan even against their rheumatologist’s recommendation, ‘I know [getting pregnant] is not the best for me…but I want to get pregnant.’	[26]
Experiencing pregnancy and parenthood	Trying to conceive	• Challenges with fertility, disease activity, and conception • Emotional impact of conception with IA	‘Two years, no child. Some of them [in-laws] are going to say, “You must take this one out, this is a barren [woman].”’	[28]
			‘With the rheumatologist advising certain medication-free timeframes before conception and the obstetrician/gynaecologist recommending surest ways to conceive on the first try, I felt like a science experiment for a while.’	[20]
	Experiencing disease activity during pregnancy & post-partum	• Perinatal flares and pregnancy complications • Perinatal medication use	‘It was the last four weeks of my pregnancy that I finally had some relief from RA but within a week of giving birth again I was in severe pain and not being able to lift my baby’.	[20]
			[One participant] said she felt very depressed after giving birth, and it was hard to know if it was a post-partum flare in her RA or post-natal depression: ‘Well they could not pinpoint it really, whether it was the flare in my arthritis or post-natal depression, all I did was cry, I felt like I was going mad.’	[15]
	Experiencing the impacts of disease on ability to be a mother	• Impacts of IA on motherhood (e.g., caring for children, breastfeeding)	Two patients reported that the disease had limited their family size, in one case preventing her from having any children.	[17]
	Making adjustments	• Impacts of IA on children • Parenting strategies	‘You get to learn to do things differently as a parent with a chronic illness. My kids know I cannot pick them up too often, so they climb up onto me for cuddles. Some days we had PJ days and they loved it as much as I did’.	[20]
	Needing support	• Childcare support • Emotional and peer support	‘I would not have been able to make this decision if I did not have the support from family and my husband’.	[20]
Navigating pregnancy and parenting with chronic disease	Worrying about unknown effects of disease on pregnancy and parenting	• Uncertainty of pregnancy progression, perinatal disease management, and ability to meet parental responsibilities	‘I’m just so nervous about taking anything it’s like my gut instinct telling me not to. If I do and something goes wrong then I’d never forgive myself you know. At the same time I do not want to end up in so much pain that I cannot care for my baby. It’s so scary.’	[29]
			‘I’m hoping and praying that none of the [my] children will get this [RA] ... because I would hate for any of them [my kids] to get arthritis. Well, you do not want them to be ill anyway, but you know, it’s a condition that’s not very kind.’	[13]
			Informants who were mothers were concerned about shortcomings in care and participation in their children’s life, and about the children’s health and chances of inheritance of rheumatic diseases.	[16]
	Struggling to cope with personal limitations	• Coping with personal limitations (e.g., ability to meet reproductive goals)	For many women, their [autoimmune rheumatic diseases] led to them feeling restrained by their physical symptoms, and they felt that they were unable to do some of the things that ‘normal’ mothers do.	[5]
			‘The sense [feeling] of not feeling entitled to something; it’s painful. It’s a bit emotional. If I cannot wash my clothes, if I cannot clean for myself or cannot carry heavy things, how is it going to be possible for me to maintain kids, you know?’	[30]
			‘I find it hard to come to terms with that. My mantra has always been, ever since I could speak as it were, that I wanted to have children. But I never had the nerve to take the initiative … There are too many obstacles and limitations, I reckon, which stop me from ever having children. Those fears run deep. I would dearly love to have children, but I do not have the courage.’	[18]
	Facing judgement from self and others	• Navigating guilt and stigma • Encountering judgement from healthcare professionals and peers	The women perform self-stigmatization in the form of self-blame and guilt when they cannot accomplish what they want in the role of mother because of fatigue, pain, or physical limitations. One of the women who had just given birth was disappointed and sorry that she did not live up to the expectations she had of herself as a future mother.	[24]
	Finding value in motherhood	• Source of purpose and motivation	‘My son and the baby that grows within me are my shining light. They are the little beings that get me out of bed in the morning when I am so stiff and sore and riddled with pain that I do not know if I can face the day’.	[20]
	Creating a self-identity beyond their disease	• Impact of self-identity (e.g., as a parent, caregiver, employee) on reproductive decisions	The women prioritize and direct the bulk of their time and energy to their working life. This is interpreted as, first and foremost, their desire to identify with the role associated with working life, rather than the identity that is solely associated with motherhood or with [rheumatoid arthritis]. The women feel that the role of worker is attractive because it gives status, and because it allows them to be self-supporting.	[24]
			The impact on women’s identity was often discussed, with women wanting to be a ‘normal’ parent and to be seen as a whole person not a disease.	[5]
Seeking information and resources for pregnancy planning	Encountering poor understanding of disease	• Feeling uninformed about pregnancy and IA management • Encountering poor understanding from family, community members, and public agencies	‘I’d say the first five years of having [rheumatoid arthritis] … I had no idea, information was scarce, I had done millions of Google searches and went to libraries and there was nothing. Nothing about pregnancy, breastfeeding and [rheumatoid arthritis]. And even my doctors were really… did not seem to know much about what medications were safe, and they’d have to go and ring other doctors, and it was really just like… I thought gee, am I the first person in the entire world to have RA and be pregnant?’	[4]
	Seeking high quality, timely, consistent, and accessible information	• Patient-specific pregnancy information (e.g., disease heredity, fertility, pregnancy complications, perinatal disease management, birth choices, and breastfeeding) • Information for early parenthood (e.g., practical strategies for managing pain/fatigue) • Information addressing prevailing misconceptions about IA	‘Some practical information as to how to do certain daily tasks without the use of certain limbs. So, you know, if my hands are so swollen that I cannot dress myself, how do I pick up my baby using my forearms, how do I…how can I carry my baby to minimise the impact on my arms and shoulders and back and wrists etc. Clothing to put the baby in…that was a big thing for me, you know, most baby clothes have… stupid press-studs, yeah, which were just the bane of my life back then. So and I managed to find clothing that was much easier for me to get on and off the baby - so that was, you know, that was a big thing. And, you know, even just…every day household tasks, you know, tips as to how to, you know, reduce your fatigue and cut down on your household duties and to…minimise your energy output’	[4]
			‘I think my problems differ from older people. I was just looking for people who work, for people with young children and how they manage their lives while having a rheumatic disease. I also wonder how I could combine work and home.’	[23]
	Seeking support services	• Avenues for emotional support (e.g., counseling; peer support groups) • Role of practical support services (e.g., social workers, hired help) • Barriers to accessing support	Some commented on the lack of resources for mothers with arthritis and desired a person or place to go to when facing specific challenges. Participants referred to 3 types of support: practical (help with child care or cleaning), emotional (help dealing with feelings), and moral (someone to listen and share). Sources of support included family and friends, health professionals, and hired help.	[14]
	Learning from peers with similar experiences	• Pragmatic information and resources • Source of emotional support and motivation • Role of online forums/support groups	Members stated that participating in the online community was a positive way for them to share concern, anxiety, or questions they had about arthritis and pregnancy.	[29]
			Discussing with others how best to talk to your children about your rheumatic disease […] Hearing from others how they involve their partner in child care […] Hearing from fellow patients how they decided whether or not to have children	[23]
Interacting with healthcare providers	Receiving care from several providers	• Value of multi-disciplinary healthcare • Barriers to patient satisfaction (e.g., care coordination/continuity, provider knowledge)	Women’s experiences varied widely, but most felt that there was a lack of well-coordinated multi-disciplinary management between different secondary care departments, as well as primary care, and this could undermine women’s trust. ‘I’ve always been the go between, the departments do not really talk to each other and I’ve many a time been in a position when I, I’ve said to either my GP [general practitioner] or my consultant you have lied to us because you are both telling me different things.’	[5]
			‘It’s confusing – the doctors from haematology told me that chloroquine is not safe for the pregnancy, I must stop it. At rheumatology the doctors said it’s OK for the baby. At haematology they told me that I must stop breastfeeding her because I must go back to the medication.’	[30]
	Discussing pregnancy planning	• Value of timely, collaborative discussions • Barriers to discussing reproductive goals (e.g., patient-provider relationship)	Most women, regardless of education level or socio-economic status, reported feeling intimidated by their rheumatologist and therefore hesitant to speak up about their true pregnancy desires.	[26]
			Younger women described feeling that they needed to get the doctor’s permission if they wanted to embark on a pregnancy.	[19]
	Seeking and receiving information	• Information on medication use and safety pre-conception and during pregnancy • Role of trust in patient-provider relationship	Focus group participants also expressed trust in their rheumatologist, but regularly sought additional sources of information (primarily from online sources) in order to make informed treatment decisions.	[4]
	Requiring compassionate and holistic care	• Approaches to compassionate patient communication • Value of provider knowledge about IA and reproductive care	Good communication, clear advice, being open to questions, compassion, kindness, understanding, encouragement, and honesty from health professionals were viewed as being important aspects of care.	[5]
**Provider perspectives**				
Providing reproductive healthcare	Understanding professional responsibility	• Rheumatologists’ role in reproductive care • Role of interdisciplinary collaboration	All rheumatologists expressed a sense of responsibility to provide some aspects of [family planning counseling and reproductive health care] to female patients of reproductive age. When asked how they defined [family planning counseling and reproductive health care], rheumatologists’ definitions unanimously centred on clarifying women’s pregnancy intentions and timing, educating patients about the associations between their diseases and pregnancy, and optimizing women’s health and anti-rheumatic drug regimens in anticipation of pregnancy.	[25]
			‘There is a tendency to say, “well, somebody else will talk to [patients] about contraception, somebody else will talk to them about family planning”.’	[25]
	Desiring more guidance	• Challenges of clinical decision-making and patient counseling for pregnancy • Need for increased knowledge about IA, pregnancy, and motherhood among healthcare providers	Rheumatologists consistently expressed that they wanted access to consensus guidelines that gave them clear recommendations for managing diseases and anti-rheumatic drugs for women before, during, after pregnancy, and through lactation.	[25]
			We suspect that these knowledge gaps and the rheumatologists’ lack of confidence in drug safety make it difficult for them to discuss medication use in pregnancy with their patients in a way that would encourage a woman to take the medication.	[26]
	Fearing negative outcomes	• Impact of rheumatologists’ fear of managing high-risk pregnancies on patient-provider relationship	Most rheumatologists could recall at least one pregnancy conceived on a teratogenic medication or during a period of high disease activity. Some were afraid of managing this complicated situation and having little control of the outcome: “That’s part of the fear, I think ... They come in crashing, and then it’s like you are responsible, and you are not sure that you are going to be able to fix that.’	[26]
	Having time restrictions	• Impact of time restrictions on reproductive care provision	Most rheumatologists expressed that competing priorities during clinic visits limited their ability to provide [family planning counseling and reproductive health care]. As one rheumatologist stated, ‘[There is] pressure to see patients in the shortest amount of time… I focus on things that only I as a rheumatologist could focus on— the disease process… there is a tendency to say, “Well, somebody else will talk to [patients] about contraception, somebody else will talk to them about family planning.’”	[25]
Interacting with patients	Discussing pregnancy planning	• Engaging patients in reproductive health discussions • Importance of understanding patients’ pregnancy intentions • Factors facilitating pregnancy planning discussions	As one rheumatologist stated, ‘When you asked me about how many pregnant patients that I have had, despite the fact that I feel pretty comfortable discussing contraception, my heart did skip a beat. And I thought, you know, it’s not one of the most pleasant things to deal with in my practice. And it’s because of the fear. There is a fear that, what if something goes wrong?... I think that we are all always concerned that anything could happen, something could go wrong…’	[25]
			Another rheumatologist noted the importance of bringing up the topic of pregnancy to aide in pregnancy planning, saying, ‘If you do not bring it up, they’ll just get pregnant.’	[26]
	Providing counseling and managing medication use	• Approaches to reproductive care and perinatal medication use counseling • Challenges managing IA pregnancies and providing medication information	Some rheumatologists use a different approach to discuss medication use in pregnancy that appealed to the patient’s desire for a healthy baby. These rheumatologists discussed medication use in pregnancy as a benefit to the baby, not primarily the mother: ‘I think they care more about the baby’s health than their own, so if I show them that there’s data that the baby actually has better outcomes in pregnancy if they are on the [medication], then they are more willing to do it. But if you say, “You’re gonna do better,” then they are not as convinced. So you have to convince them that it’s better for the baby.’	[26]
	Building the patient-provider relationships	• Challenges building patient-provider relationships • Role of patient trust in adherence to medical advice	Another rheumatologist described how feelings of fear and anxiety fractured the relationship with a newly pregnant patient, ‘I think I wasn’t able to build up a very good physician-patient relationship, because I think that I got so scared that I kind of blurted out all the data for every single one of the drugs that we were talking about. I think that kind of scared her [patient]. She did not follow up very well… the pregnancy went well, but she never really did the follow-up as well as she should, and I felt it was because she wasn’t trusting my judgment.’	[25]
			One rheumatologist described the patient dilemma when receiving care for their disease during pregnancy as, ‘does this person want to hurt me and my baby, or do they want to help me and the baby?’	[26]
Coordinating patient care with other providers	Collaborating on patient care planning	• Value of multi-disciplinary collaboration • Barriers to patient care coordination	As one rheumatologist described: ‘Rheumatology and OB-GYN [obstetrics-gynaecology], it should be a collaboration. I do not think it’s reasonable to expect family practitioners [to manage reproductive care] when they manage so many diseases and so many medications.’	[25]
			‘It’s actually getting everybody that might need to be involved to see it in a more holistic way.’	[5]
	Providing support services	• Role of tailored support services for IA (e.g., pre-conception counseling, social/psychological supports)	Health professionals also felt that it was important to consider the specific support needs of women with [autoimmune rheumatic diseases]. ‘I’m just thinking about the, there’s very specific anxiety and concerns that come for these women in the context of their parenting role having a chronic sort of autoimmune disorder.’	[5]

### Patient perspectives

#### **Making decisions about planning and experiencing pregnancy**

Making decisions about having children comprised a dynamic balance of patients’ reproductive desires, the impact of disease on their ability to experience pregnancy, and the availability of information to guide preparedness. We identified 5 descriptive themes related to patients’ reproductive decision-making in 17 studies [4, 5, 13, 14, 16, 17, 19–24, 26–30].

When discussing *making decisions about becoming pregnant*, patients emphasized the role of their disease within this process [5, 13, 16, 17, 19–24, 26, 29, 30]. Many expressed concerns about disease activity and management during pregnancy and post-partum [5, 24, 29, 30] and shared fears of being unable to care for a child due to their IA [20, 21, 24, 29, 30]. Some patients expressed strong desires to bear children despite these concerns [20, 26, 29, 30] or sought information about alternatives to childbearing (e.g., adoption) [5, 16, 29]. Others chose not to have children or limited their family size [17, 18, 20, 21, 23, 24, 29, 30]. Factors influencing childbearing decisions included their medications [5, 17, 20, 22, 29], disease severity [5, 17, 20, 30], previous pregnancy experiences [20, 30], and support from partners, family, and healthcare providers [5, 19, 20, 30]. Patients in one study discussed navigating contraception planning and pregnancy termination [30].

*Making decisions about using medications* [4, 5, 13, 14, 20, 22, 26, 27, 29] and *breastfeeding* [5, 20] were closely intertwined with reproductive decisions. Patients reported desiring more information about medication safety related to fertility, pregnancy, and breastfeeding [4, 5, 20, 29], consequences of changing or discontinuing medications on disease activity and subsequent medication efficacy [4, 5, 20, 29], alternative or adjunct (e.g. folic acid supplementation) therapies [4, 29], and lifestyle modifications [4, 29]. Factors influencing medication decisions included concerns about medication impacts on foetal development [29] and medication discontinuation on disease management and pregnancy [20, 27] as well as desiring to experience pregnancy [26] and breastfeeding [5, 20]. Sources of medication information comprised healthcare providers, including rheumatologists, nurses, and pharmacists [4, 26, 27, 29], as well as online sources [26, 29]. Patients indicated desiring more decisional support related to medication use, particularly guidance from providers [4, 5].

Patients recounted *needing to prepare for pregnancy and parenthood*, including timing conception, making medication adjustments, achieving low disease activity, and recognizing potential complications (e.g., miscarriage) [5, 16, 20, 27, 29, 30]. Patients also identified needing to prepare for parenting while managing post-partum flares and establishing support systems that include family members and mothers with similar experiences [5, 20, 29].

Many patients described having to balance *prioritizing the needs of mother and child* [4, 20, 24, 26, 28–30]. Patients shared grappling with trade-offs between their desires to become pregnant and breastfeed and to avoid potential harms from medication use, disease flares, and permanent joint damage [4, 26, 29]. They reported self-advocacy was instrumental to realizing their reproductive goals [4, 28–30].

#### **Experiencing pregnancy and parenthood**

Patients’ experiences of pregnancy and early parenthood were intertwined with their disease management, expectation of pregnancy and parenting, and ability to access support, which was captured by 5 descriptive themes in 16 studies [4, 5, 13–15, 17–21, 24, 26–30].

Patients reported challenges when *trying to conceive* related to fertility, disease activity, and pressure to conceive quickly after discontinuing certain medications [13, 15, 18–20, 28, 30]. These were noted to be sources of stress, fear, and emotional hardship [18, 20, 28, 30]. Some patients felt they needed permission from their provider to conceive [19, 28]. Many reported *experiencing disease activity during pregnancy and post-partum*, describing worsening symptoms, limitations preforming daily tasks, requiring support from caregivers, and experiencing pregnancy complications and hospitalization [4, 13–15, 20, 27, 29, 30]. For some, these hardships impeded their ability to work and posed financial challenges [20, 29]. While some patients resolved not to take medications perinatally despite experiencing disease-related challenges [14, 29], others described needing to use medications due to disease severity or worsening symptoms [4, 20, 29]. Some patients experienced disease remission during pregnancy [14, 20, 29]. Several patients who restarted medications post-partum found they were no longer effective and had to seek new therapies [20].

Patients described *experiencing the impacts of disease on ability to be a mother*, which included navigating pain and fatigue, difficulty physically caring for children, and being unable to breastfeed due to physical limitations or medications used for post-partum flares [5, 14, 15, 17, 20, 21, 24, 28, 29]. Patients reported *making adjustments* to accommodate for personal disability [5, 13–15, 20, 24, 29]. Parenting strategies included choosing to stay at home or arranging flexible work hours, prioritizing rest and self-care, planning family activities requiring limited physical activity, and teaching children to self-sooth [14, 20, 24, 29]. Some patients reported pride in raising highly independent children, although this was also a source of guilt [14, 20]. Ultimately, patients reported *needing support* for the practical and physical demands of pregnancy and parenthood [5, 13, 14, 17, 20, 21, 26, 29]. Many depended on support enlisted from extended family, while others received limited assistance [5, 13, 14, 17, 20, 21, 29]. Additionally, patients valued emotional and peer support from online communities [5, 26, 29].

#### **Navigating caregiving with chronic disease**

Reproductive uncertainty related to IA placed a heavy burden on patients’ emotional and psychological wellbeing. We identified 5 descriptive themes about the impacts of being a caregiver with a chronic debilitating disease in 17 studies [4, 5, 13–20, 23, 24, 26–30].

Many patients reported *worrying about unknown effects of disease on pregnancy and parenting* [4, 5, 13, 15–18, 20, 23, 24, 27–30]*.* Patients struggled with uncertainty related to managing their disease activity during pregnancy and post-partum [4, 15, 16, 20, 28–30]. They shared concerns about fertility [5, 18, 28, 30], timing conception [5, 20], passing their disease to their children [5, 13, 16–18, 23, 30], experiencing pregnancy complications [5, 17, 18, 27, 28, 30] or disease progression [5, 18, 20, 24], causing foetal harm with medication use [18, 20, 29], and being unable to meet their parental responsibilities (e.g., taking care of children, financially providing for family) [5, 16, 18–20, 29]. Accordingly, patients described the emotional impacts of *struggling to cope with personal limitations* related to their disease [4, 5, 15, 19, 20, 24, 28–30]. Patients felt isolated, anxious, frustrated, beaten down, and useless [4, 5, 15, 18, 20, 28–30] as well as restricted in their ability to meet their reproductive goals [18, 30].

Patients described *facing judgement from self and others* [4, 14–16, 20, 24, 26, 28, 30]. They associated stigma with their disease, describing IA as an ‘*invisible disability*’ [24]. They recounted feeling guilty for requiring help at home and accommodations at work, as well as their children having to develop independence at an early age [15, 16, 20, 24]. Patients shared experiencing judgement and lack of understanding from healthcare professionals regarding their pregnancy intentions, medication decisions, and uptake of medical advice [4, 14, 26] as well as members of their community regarding their ability to become pregnant and fulfil their parental responsibilities [15, 28, 30].

Despite challenges, patients described *finding value in motherhood* as a driving force for pursing pregnancy and their children as a source of purpose and motivation [5, 15, 20, 24, 28, 29]. Additionally, they depicted *creating a self-identity beyond their disease* as a parent, caregiver, and employee [5, 13, 15, 20, 24]. Patients who had children wanted to be seen as ‘*normal*’ parents [5, 15], while those who perceived parenthood was not in their best interest found other goals to pursue [20].

#### **Seeking information and resources for pregnancy planning**

Regarding knowledge gaps concerning pregnancy planning with IA, we identified 4 descriptive themes related to seeking information about pregnancy planning and disease management in 11 studies [4, 5, 13–15, 20, 23, 26, 28–30].

Patients reported *encountering poor understanding of disease* personally and from their family, community members, and public agencies providing support resources [4, 14, 20, 28, 30]. Patients voiced feeling uninformed and receiving limited information about pregnancy and disease management from healthcare providers [4, 5, 15, 20, 30]. Overwhelmingly, patients shared challenges when *seeking high quality, timely, consistent, and accessible information* about perinatal disease management [4, 5, 15, 23, 26, 29, 30], emphasizing the importance of specific and tailored information given maternal stage and health literacy [4, 5, 23, 29]. Prior to pregnancy, patients wanted to receive information about disease heredity, fertility, pregnancy planning, pregnancy complications, perinatal disease management (e.g., medication use and discontinuation), birth choices (e.g., natural birth, caesarean), and breastfeeding [4, 5, 15, 23, 29]. Additionally, patients emphasized wanting information and support for early parenting related to pain and mobility, including practical strategies for adjusting to daily challenges [4, 5, 23]. Most reported seeking written information that can be accessed electronically [4, 15, 26, 29]. They also identified needing information addressing prevailing misconceptions about IA to be available for family, friends, employers, and public agencies [4, 5, 30].

Relatedly, patients described *seeking support strategies and services* [4, 5, 13, 14, 23, 29]. Patients emphasized wanting more avenues for emotional support, including counseling and peer support groups, for addressing feelings of uncertainty and isolation [4, 5, 14, 29]. Patients depicted the role of practical support services, such as social workers, nurses, and hired help, for assisting in household tasks, childcare, and activities of daily living [4, 5, 13, 14, 23]. Patients also described seeking practical strategies for minimizing pain and fatigue when caring for a baby (e.g., bathing, dressing, and feeding tasks) and information about assistive devices [4, 5, 14]. Barriers to accessing support were regional variability of available services, travel requirements for specialized services, existing services not accounting for the needs of patients with IA (e.g., perinatal classes that are physically strenuous), and receiving continuity of care [5].

Finally, patients expressed valuing *learning from peers with similar experiences* through shared personal concerns, experiences, and advice [4, 5, 23, 26, 29]. Patients were interested in pragmatic information and resources used by peers and further identified peers as sources of emotional support and motivation [4, 23, 29]. The majority of patients participated in online forums or support groups [5, 26, 29].

#### **Interacting with healthcare providers**

We identified 4 descriptive themes in 9 studies related to patients’ experiences interacting with healthcare providers [4, 5, 14, 15, 20, 26, 28–30].

Patients discussed their experiences *receiving care from several providers,* including rheumatologists, primary care physicians, obstetricians, counselors, physiotherapists, occupational therapists, midwives, and pharmacists [4, 5, 20, 29, 30]. They valued receiving care from a multi-disciplinary team when possible [5, 29]. Patients who reported dissatisfaction with their care team mentioned issues of inadequate care coordination, a lack of care continuity, and a high frequency of appointments [5]. Some described receiving advice and encountering judgement from allied health professionals with poor disease understanding [4, 5, 14, 30].

Patients also shared diverse experiences *discussing pregnancy planning* with their providers [4, 5, 14, 15, 20, 26, 29]. They emphasised desiring timely and collaborative conversations about pregnancy planning [5, 15, 26]. Patients who reported positive patient-provider relationships shared establishing a plan for when they decide to start trying to conceive [29]. Those who reported concerns discussing their reproductive goals with providers shared feeling intimidated, fearing judgement, wanting to avoid confrontation, and not receiving support for becoming pregnant [5, 14, 15, 26, 29].

Nevertheless, patients turned to their healthcare providers when *seeking and receiving information* about pregnancy planning [4, 5, 15, 26, 29, 30]. Information from providers predominantly pertained to medication use and safety pre-conception and during pregnancy [29]. Rheumatologists were identified as the primary source of medical information, although some patients also obtained information from online sources, including forums and social media [4]. Trust in their provider influenced whether patients decided to follow providers’ recommendations or seek information from other sources [4, 26]. Factors influencing provider trust included the amount of information shared about pregnancy with IA, the consistency of information received from their healthcare team, their information preferences, and the provider’s confidence as perceived by the patient [4, 5, 30].

Overwhelmingly, patients indicated *requiring compassionate and holistic care* from providers [5, 14, 15, 28, 29]. Patients valued providers who were educated in their disease, particularly relating to reproductive care, supported their health and reproductive goals, and provided proactive care based on their individual patient needs beyond medication use [5, 14, 15, 28, 29].

### Provider perspective

#### **Providing reproductive health care**

Reproductive care provision was influenced by the provider’s perceived professional responsibility for meeting their patients’ reproductive goals, their fears of responsibility for negative outcomes, and their capacity to incorporate reproductive care into their practice. We identified 4 descriptive themes related to the provider’s perspective of delivering reproductive care in 4 studies [4, 5, 25, 26].

Providers, primarily rheumatologists, discussed *understanding their professional responsibility* [5, 25]. Overall, rheumatologists believed providing some reproductive care was within their professional responsibility, including clarifying pregnancy intentions, educating patients about pregnancy with IA, and optimizing patient’s medication and disease management in anticipation of pregnancy [25]. This definition did not include contraception or abortion care [25]. While rheumatologists recognized it was their responsibility to ensure reproductive age patients on teratogenic medications were using contraception, they focused on managing their disease [25]. They preferred that primary care physicians or gynaecologists prescribe contraceptives [25]. Generally, providers agreed pre-conception counseling was necessary for patients with IA; however, they were unclear about which profession should provide this service [5].

Providers emphasised *desiring more guidance* for providing reproductive care [5, 25, 26]. Rheumatologists reported that clinical decision-making and patient counseling was challenging given gaps in clinician knowledge about medication safety in pregnancy [26]. They consistently expressed wanting consensus guidelines with clear recommendations for managing diseases and medication use for patients before, during, and after pregnancy and for breastfeeding [25]. Both providers and patients recognized the need for increased awareness of IA and its impact on pregnancy and motherhood among healthcare providers caring for IA patients [5].

Notably, rheumatologists described the impacts of *fearing negative outcomes* on their patients [25, 26]. Most rheumatologists described experiencing tension between respecting patients’ autonomy to become pregnant and fear of managing high-risk pregnancies and having limited control of pregnancy outcomes [25, 26]. Many had difficulty understanding why patients chose to pursue pregnancy given their risk of complications [26]. Some rheumatologists recognized their own fear may influence how they counsel patients [25]. Additionally, *having time restrictions* was reported as a barrier to providing reproductive care [4, 25, 26].

#### **Interacting with patients**

Providers’ experiences interacting with patients were influenced by their abilities to hold space for patient’s desires amidst their personal beliefs, to harness trust, and to engage patients in discussions about reproductive health, which was captured by 3 descriptive themes in 3 studies [5, 25, 26].

Providers shared their experiences *discussing pregnancy planning* with their patients [5, 25, 26]. Some rheumatologists recognized they may overestimate the magnitude of pregnancy risks and that their caution may discourage patients from discussing their reproductive goals and place undue pressure on patients to avoid pregnancy [25]. As pregnancy needs to be planned carefully with IA, rheumatologists acknowledged the importance of being aware of patients’ pregnancy intentions and usually respected their patients’ autonomy to pursue pregnancy [5, 25, 26]. Factors facilitating pregnancy planning discussions included patient initiation, reproductive decisiveness, and having a female provider [25, 26].

Providers also discussed *providing counseling and managing medication use* [5, 25, 26]. Rheumatologists felt most comfortable discussing medications for perinatal disease management [25]. Some recommend pre-natal folic acid supplementation; however, most felt uncomfortable prescribing contraceptives [25]. Providers described medically ill-timed pregnancies (i.e., conception while taking a teratogenic medication or experiencing high disease activity) and medication non-adherence during pregnancy as dilemmas in care [26]. Noted challenges of providing medication information were communicating medication risk to patients, knowledge gaps about medication safety in pregnancy, and confidence in available data [25, 26]. Given the recognized desire to avoid foetal harm among patients, some rheumatologists noted the value of framing discussions about medication use as a benefit to both mother and baby [26]. Overall, providers underlined needing to offer patients pre-conception counseling and timely high quality written information for pre-conception, pregnancy, and post-partum [5].

Finally, providers discussed challenges of *building patient-provider relationships* [5, 25, 26]. Several rheumatologists acknowledged that a ‘*gloom and doom*’ approach towards pregnancy planning diminished patient trust [25, 26]. Trust was recognized to contribute to patient adherence to medical advice [26]. Distrust of the medical community was noted to persist in marginalized communities [26].

#### **Coordinating patient care with other providers**

We identified 2 descriptive themes related to coordinating patient care in 3 studies [5, 25, 26].

Providers, like patients, recognized the value of *collaborating on patient care planning* with a multi-disciplinary team, including obstetrician-gynaecologists, nurses, midwives, and occupational therapists [5, 25, 26]. Key barriers to patient care coordination included limited communication between providers and regional infrastructure for specialized multi-disciplinary teams, leading to unnecessary lab testing and inconsistent medication recommendations [5, 26].

Moreover, providers recognized the need for *providing support services* and tailored care to IA patients [5, 25]. Identified services included pre-conception counseling, social and psychological supports for the emotional impacts of high-risk pregnancy and parenting with IA, and social support for the practical challenges of pregnancy and early parenting [5, 25].

## Discussion

We thematically synthesized 20 qualitative studies on the pregnancy and early parenting experiences of 368 patients with IA and 51 providers. Our conceptual model illuminates the complex relationships between patient and provider perspectives about reproductive care provision, patients’ experiences of IA and pregnancy, and patients’ processes for seeking information and making reproductive decisions. Our analysis suggests how informed patients felt, how well their IA was managed, and how capable they were of accessing support informed the acceptability of their pregnancy and early parenting experiences.

For providers, our results illuminate the necessity of collaborative, comprehensive, and patient-centred approaches to delivering reproductive care. There is room for improving how providers build trust, support patients’ reproductive goals and expectations, and collaborate in making decisions, while in turn building their professional confidence in reproductive care provision to patients with IA [5, 14, 15, 25, 26, 28, 29]. Moreover, patients would benefit from comprehensive care beyond medication use for IA through multi-disciplinary care coordination of their healthcare team, connecting to peers with similar experiences, and receiving referrals to psychological and social services and resources [4, 5, 14, 25, 26, 29].

For patients, our review highlights measures within a patient’s control with the potential to improve their reproductive care experience. It is important that patients have conversations with their providers about pregnancy planning early to reduce potential hardships related to achieving low disease activity pre-conception, timing pregnancy, making medication adjustments, and preparing for potential complications [5, 20, 29]. As such, patients must feel comfortable discussing pregnancy planning and their pregnancy intentions with providers. When seeking a referral for alternative or additional support, patients may consider asking potential providers about their knowledge and experience providing reproductive care to patients with IA. Beyond clinical disease management, patients may benefit from practical and emotional support services to mitigate the personal impacts of pregnancy and parenting with IA [5, 25].

It is important to contextualize our review with the release of perinatal guidelines for medication use in IA in 2016 and 2020 [31–34]. We included articles published from 2006 to 2020. Studies specifically exploring pregnancy [4, 5, 20, 25, 26, 29, 30], as opposed to parenting with IA, first appeared in 2012 with the majority published between 2018 and 2020. This included few studies (*n* = 3) on the provider perspective [5, 25, 26], which depicted the need for professional guidance for rheumatologists providing reproductive care, including recommendations for managing perinatal disease activity and medication use, despite the release of aforementioned guidelines.

Strengths and limitations of our review warrant discussion. We developed and employed a comprehensive search with research librarians to capture published studies reporting on pregnancy and early parenting among female patients with IA. We used established methods for evaluating reporting of qualitative studies and synthesizing qualitative results [9, 11, 12], which provided transparency of our data analysis and interpretation processes. An inductive approach ensured themes were derived from the data and two authors reviewed the coding framework and thematic synthesis to enhance the credibility of findings. Some studies provided limited participant demographic information, which may affect the adaptability of our results. It is also possible that the identification of relevant studies was limited by publication bias; however, as qualitative synthesis aims to provide interpretive explanation rather than predictions, sampling is meant to be purposive as opposed to exhaustive [12, 35]. Finally, our analysis was limited by the scope of research in this area, which to-date has not extensively explored the intersectional influences of gender and race on patient’s reproductive experiences or their experiences managing contraception and terminating pregnancies.

Our review provides a conceptional understanding of the reproductive care experiences of female patients with IA and their providers. It is critical for providers to recognize the toll of uncertainty felt by patients ‘*walking into the unknown’* of navigating pregnancy and early parenthood. Enhancing the degree of support felt by individuals with IA by engaging in open conversations about pregnancy planning and shared decision-making may empower patients and strengthen patient-provider relationships. Interventions focused on facilitating access to reliable and timely information and practical and emotional support may help lessen the personal impacts of pregnancy planning and parenting with IA.

## Supplementary Information

**Additional file 1.**

## Data Availability

Data sharing is not applicable to this article as no datasets were generated or analysed for the study.

## Abbreviations

AS: Ankylosing spondylitis
CASP: Critical Appraisal Skills Programme
ENTREQ: Enhancing transparency in reporting the synthesis of qualitative research
GP: General practitioner
IA: Inflammatory arthritis
JIA: Juvenile idiopathic arthritis
OB-GYN: Obstetrics-gynaecology
PsA: Psoriatic arthritis
RA: Rheumatoid arthritis
SLE: Systematic lupus erythematosus
