# Supplementary material for: “Walking into the unknown…” key challenges of pregnancy and early parenting with inflammatory arthritis: a systematic review and thematic synthesis of qualitative studies
Source: Arthritis Res Ther. 2021 Apr 21;23:123. doi: 10.1186/s13075-021-02493-z (PMC8059168; doi:10.1186/s13075-021-02493-z)
Supplement: Supplementary file 1 — Additional file 1. [file 13075_2021_2493_MOESM1_ESM.docx]

**Additional file 1**

| **Table 1.** Database(s): Ovid MEDLINE(R) and Epub Ahead of Print, In-Process & Other Non-Indexed Citations, Daily and Versions(R) 1946 to Feb 01, 2020. | | | |
| --- | --- | --- | --- |
| **Line** | **Searches** | **Results** | |
| 1. | rheumatic diseases/ or arthritis, juvenile/ or exp arthritis, rheumatoid/ | 139649 | |
| 2. | exp Lupus Erythematosus, Systemic/ | 58630 | |
| 3. | arthritis, psoriatic/ or spondylitis, ankylosing/ | 19655 | |
| 4. | (arthritis adj2 (psoriatic or juvenile)).mp. | 22257 | |
| 5. | (rheumat* or lupus or ankylosing spondylitis or SARD*).mp. | 298443 | |
| 6. | or/1-5 | 321136 | |
| 7. | (pregnan* or prenatal or antenatal or perinatal).mp. | 1045240 | |
| 8. | Family Planning Services/ | 24583 | |
| 9. | exp Contraception/ | 26461 | |
| 10. | exp Infertility/ | 64843 | |
| 11. | exp Pregnancy Complications/ | 418900 | |
| 12. | (family planning or contraception or fertility or infertility or miscarriage*).mp. | 232897 | |
| 13. | mother*.mp. or mothers/ | 233119 | |
| 14. | or/7-13 | 1335661 | |
| 15. | grounded theory/ or qualitative research/ | 52189 | |
| 16. | (qualitative* or focus group* or interview* or mixed method* or mixed-method* or content analysis or thematic analysis or phenomenological study or ethnograph* or interpretive description or narrative* or semi-structured or unstructured or face-to-face or constant comparative or participant observation or audio recorded).mp. | 660999 | |
| 17. | px.fs. | 1032241 | |
| 18. | or/15-17 | 1526615 | |
| 19. | 6 and 14 and 18 | 367 | |
| **Table 2.** Database(s): Embase 1974 to Feb 01, 2020. | | |  |
| **Line** | **Searches** | **Results** |  |
| 1. | rheumatic disease/ or exp rheumatoid arthritis/ | 139649 |  |
| 2. | systemic lupus erythematosus/ | 58630 |  |
| 3. | psoriatic arthritis/ or ankylosing spondylitis/ | 19655 |  |
| 4. | (arthritis adj2 (psoriatic or juvenile)).mp. | 22257 |  |
| 5. | (rheumat* or lupus or ankylosing spondylitis or SARD*).mp. | 298443 |  |
| 6. | or/1-5 | 321136 |  |
| 7. | (pregnan* or prenatal or antenatal or perinatal).mp. | 1045240 |  |
| 8. | Family Planning/ or fertility/ | 24583 |  |
| 9. | exp Contraception/ | 26461 |  |
| 10. | exp Infertility/ | 64843 |  |
| 11. | exp Pregnancy Complication/ | 418900 |  |
| 12. | Mother/ |  |  |
| 13. | (family planning or contraception or fertility or infertility or miscarriage*).mp. | 232897 |  |
| 14. | or/7-13 | 1537654 |  |
| 15. | grounded theory/ or qualitative research/ | 75672 |  |
| 16. | (qualitative* or focus group* or interview* or mixed method* or mixed-method* or content analysis or thematic analysis or phenomenological study or ethnograph* or interpretive description or narrative* or semi-structured or unstructured or face-to-face or constant comparative or participant observation or audio recorded).mp. | 841688 |  |
| 17. | or/15-16 | 842006 |  |
| 18. | 6 and 14 and 17 | 557 |  |

| **Table 3.** Database(s): CINAHL (EBSCO) to Feb 01, 2020. | | |
| --- | --- | --- |
| **Line** | **Searches** | **Results** |
| S1 | (MH "Rheumatic Diseases") OR (MH "Arthritis, Rheumatoid+") | 29,928 |
| S2 | (MH "Lupus Erythematosus, Systemic+") | 8,255 |
| S3 | (MH "Spondylitis, Ankylosing") OR (MH "Arthritis, Psoriatic") | 4,889 |
| S4 | arthritis n2 (psoriatic or juvenile) | 6,084 |
| S5 | rheumat* or lupus or ankylosing spondylitis or sard* | 53,293 |
| S6 | S1 OR S2 OR S3 OR S4 OR S5 | 56,748 |
| S7 | pregnan* or prenatal or antenatal or perinatal or mother* | 293,666 |
| S8 | (MH "Family Planning+") | 10,596 |
| S9 | (MH "Contraception+") | 9,691 |
| S10 | (MH "Infertility") | 10,608 |
| S11 | (MH "Pregnancy Complications+") or (MH "Mothers+") | 116,576 |
| S12 | "family planning" or contraception or fertility or infertility or miscarriage* | 44,108 |
| S13 | S8 OR S9 OR S10 OR S11 OR S12 | 159,031 |
| S14 | (MH "Qualitative Studies+") | 134,492 |
| S15 | qualitative* or "focus group*" or interview* or "mixed method*" or mixed-method* or "content analysis" or "thematic analysis" or "phenomenological study" or ethnograph* or "interpretive description" or narrative* or semi-structured or unstructured or face-to-face or "constant comparative" or "participant observation" or "audio recorded" | 421,371 |
| S16 | S14 OR S15 | 430,172 |
| S17 | S6 AND S13 AND S16 | 34 |

| **Table 4.** Database(s): PsycInfo (EBSCO) to Feb 01, 2020. | | |
| --- | --- | --- |
| **Line** | **Searches** | **Results** |
| S1 | (DE "Rheumatoid Arthritis") OR (DE "Lupus") | 2,621 |
| S2 | arthritis n2 (psoriatic or juvenile) | 467 |
| S3 | rheumatic or rheumatoid or lupus or ankylosing spondylitis or sard* | 6,465 |
| S4 | S1 OR S2 OR S3 | 6,663 |
| S5 | pregnan* or prenatal or antenatal or perinatal | 78,801 |
| S6 | DE "Family Planning" OR DE "Birth Control" OR DE "Delayed Parenthood" OR DE "Infertility" OR DE "Sterility" OR DE "Obstetrical Complications" OR DE “Mothers” | 50,287 |
| S7 | "family planning" or contraception or fertility or infertility or miscarriage* or mother* | 150,556 |
| S8 | S5 OR S6 OR S7 | 202,354 |
| S9 | DE "Qualitative Methods" OR DE "Focus Group" OR DE "Grounded Theory" OR DE "Interpretative Phenomenological Analysis" OR DE "Narrative Analysis" OR DE "Semi-Structured Interview" OR DE "Thematic Analysis" OR DE "Qualitative Measures" | 14,454 |
| S10 | qualitative* or "focus group*" or interview* or "mixed method*" or mixed-method* or "content analysis" or "thematic analysis" or "phenomenological study" or ethnograph* or "interpretive description" or narrative* or semi-structured or unstructured or face-to-face or "constant comparative" or "participant observation" or "audio recorded" | 596,271 |
| S11 | S9 OR S10 | 596,895 |
| S12 | S4 AND S8 AND S11 | 39 |

| **Table 5.** Database(s): Social Sciences Citation Index & Web of Science Core Collection (Clarivate)  Feb. 01, 2020. Indexes=SCI-EXPANDED, SSCI, A&HCI, CPCI-S, CPCI-SSH, ESCI Timespan=1900-2020. | |
| --- | --- |
| **Searches** | **Results** |
| TS=((arthritis NEAR/2 (psoriatic or juvenile) or (rheumat* or lupus or ankylosing spondylitis or SARD*)) AND  (pregnan* or prenatal or antenatal or perinatal or "family planning" or contraception or fertility or infertility or miscarriage* or mother*)  AND  (qualitative* or focus group* or interview* or mixed method* or mixed-method* or content analysis or thematic analysis or phenomenological study or ethnograph* or interpretive description or narrative* or semi-structured or unstructured or face-to-face or constant comparative or participant observation or audio recorded)) | 349 |
